# A spiking model of superior colliculus for bottom-up saliency

**DOI:** 10.1186/1471-2202-14-S1-P185

**Published:** 2013-07-08

**Authors:** Micah Richert, Jayram Moorkanikara Nageswaran, Sach Sokol, Botond Szatmary, Csaba Petre, Filip Piekniewski, Eugene Izhikevich

**Affiliations:** 1 Brain Corporation, San Diego, CA 92121, USA; 2 Mind Brain Institute, John Hopkins University, Baltimore, MD 21218, USA

## 

Active exploration of visual space is controlled by a saccadic system using a combination of bottom-up and top-down signals. Rate based models [1] and simple V1 models (e.g. [2]), have been proposed to explain bottom-up or "pop-out" attentional mechanisms. We present a biologically detailed spiking model of superior colliculus to generate saccadic eye movements using a competitive mechanism based on spike arrival times. Various pop-out tasks based on color, orientation features and motion is used to analyze the reaction time of the model.

The superior colliculus is modeled as a two layer network with feature-specific superficial layers driving a common deep layer. The superficial layers of the superior colliculus are topologically organized and is driven by retinal parasols cells, V1 layer 2/3 orientation-selective cells, V1 layer 2/3 color-selective cells, and MT cells. These enable luminance, orientation, color and motion based popout respectively. In this model, the earliest spikes are considered the most salient. A saccade is triggered when there is a consistent winner in the superficial layers at the same spatial region over an extended period of time, 40 ms to 100 ms. The superficial layers implement a temporal winner-take-all mechanism; specifically, there exists a separate inhibitory subnetwork that allows each superficial layer to choose a winner. The deep layer integrates these winners over time and selects a single spatial region. It implements a bump attractor network mediated by short-range excitation (AMPA and NMDA) and long range inhibition, which generates a bump of activity before and during a saccade. An interesting property of bump attractor network based circuit is that a single cell response is independent of size of the stimulus. Similar spiking response was also observed in most of the visually selective superior colliculus cells in monkeys [3]. Additionally, this model captures known behavioral and neurophysiological properties of the superior colliculus such as saccadic suppression, activity buildup before saccade generation, and GABAb mediated inhibition from the deep layer to superficial layers resulting in selective inhibition in recently attended region. All the connections in the SC model are pre-wired to satisfy the response characteristics of superficial and deep layers.

The reaction time of the model was tested on simple and conjunctive pop-out tasks. We observe only a mild shift in reaction time based on the number of distractors. Higher reaction time was observed as the complexity of the search task was increased. Also, reaction time increases based on the level of visual hierarchy that is connected to the superior colliculus layers.

## Conclusion

A spiking model of superior colliculus is presented that demonstrate many interesting dynamics observed in superficial and intermediate layers of the superior colliculus [3]. A simple reaction time analysis of the model showed latency ranges that match the hierarchy of the incoming visual layers, with simple features eliciting much faster response than more complex features.
